# Nicotine-related interpretation biases in cigarette smoking individuals

**DOI:** 10.1038/s41598-024-55256-6

**Published:** 2024-02-27

**Authors:** Alla Machulska, Marcella L. Woud, Julia Brailovskaia, Jürgen Margraf, Tim Klucken

**Affiliations:** 1 Department of Clinical Psychology and Psychotherapy, Faculty of Psychology, University of Siegen, Siegen, Germany; 2 Department of Clinical Psychology and Experimental Psychopathology, Faculty of Psychology, Georg-August-University, Göttingen, Germany; 3 Mental Health Research and Treatment Center, Faculty of Psychology, Ruhr-University Bochum, Bochum, Germany; 4 DZPG (German Center for Mental Health), Partner Site Bochum/Marburg, Bochum, Germany

**Keywords:** Human behaviour, Psychology, Health care

## Abstract

Addictive behaviors are characterized by information processing biases, including substance-related interpretation biases. In the field of cigarette smoking, such biases have not been investigated yet. The present study thus adopted an open-ended scenario approach to measure smoking-related interpretation biases. Individuals who smoke, those who ceased smoking, and those without a smoking history (total sample *N* = 177) were instructed to generate spontaneous continuations for ambiguous, open-ended scenarios that described either a smoking-related or neutral context. Overall, people who smoke generated more smoking-related continuations in response to smoking-relevant situations than non-smoking individuals or people who had stopped smoking, providing evidence for a smoking-related interpretation bias. When differentiating for situation type within smoking-relevant scenarios, smoking individuals produced more smoking-related continuations for positive/social and habit/addictive situations compared to negative/affective ones. Additionally, the tendency to interpret habit/addictive situations in a smoking-related manner was positively associated with cigarette consumption and levels of nicotine dependence. Exploratory analyses indicated that other substance-related continuations were correlated with their respective behavioral counterparts (e.g., the level of self-reported alcohol or caffeine consumption). The present study is the first to demonstrate smoking-related interpretation biases in relation to current cigarette smoking. Future studies should investigate the causal role of such biases in the initiation and/or maintainance of nicotine addiction and the merit of Interpretation-Bias-Modification training to support smoking cessation.

## Introduction

People differ in how they interpret situations and this in turn is related to both emotional and behavioral responses. That is, the same situation can activate different interpretations, depending on past experiences or motivational states. Most importantly, these interpretations can be either explicit and deliberate, or operate fast and automatic on an implicit level (e.g.,^1^). Explicit and implicit interpretations do not always have to coincide, and in fact a central hallmark of many psychological disorders, especially addictive disorders, is a mismatch between reflective and automatically-triggered cognitive processes^2^. More specifically, dual process models of addiction [e.g., 1] postulate that individuals suffering from substance-use disorders are particularly characterized by biased implicit cognitive processes. Such processes involve the tendency to process disorder-relevant stimuli in a systematically biased manner, for example to focus primarily on addiction-related cues rather than on other, more neutral stimuli in the environment. Cognitive biases include e.g., biased attentional processing and behavioral tendencies, respectively, as well as interpretational biases. Furthermore, such biases may become spontaneously activated in various circumstances and may therefore become a major driving force for continued substance use despite opposite intentions^3^ (but for a critical discussion on dual process accounts, see^4,5^). In this context, nicotine addiction is of special importance as cigarette smoking continues to be one of the most frequent substance-use disorders worldwide^6^, leading to immense and detrimental consequences for individual health as well as health care systems. Notable, even if most smoking individuals want to quit^7^, supported by e.g., having planned intentions, the majority does not succeed even after gold-standard treatment^8–10^. It is conceivable that implicit cognitive processes, including substance-related interpretation biases, can interfere with explicit processes such as long-term goals and self-regulation^2^. Therefore, investigating the role of biased interpretations is crucial for both understanding nicotine addiction and promoting effective treatment programs.

In recent years, different paradigms have been developed to access biased interpretations and their associations with emotional disorders and addictions. For instance, the Implicit-Association-Task (IAT;^11^) and its various adaptations (i.e., wanting-IAT:^12^; approach-avoid single-target-IAT:^13,14^) have been developed to measure implicit associations. By doing so, it has been shown that positive (but not negative) implicit smoking associations predict smoking behavior over and above explicit measures (e.g.,^15^). However, null-findings have also been reported^14,16,17^, and reaction time (RT)-based paradigms are not without limitations, e.g., they have been criticized for insufficient psychometric properties in terms of both reliability and validity^18^. Hence, alternative paradigms were needed and open-ended scenario tasks provided a promising route in this context (e.g.,^19,20^; and for an overview, see^21^). Such tasks require participants to give their first, spontaneous interpretation of ambiguous cues such as single words, simple scenarios, or word pairs^21^. For example, word association tasks require participants to give their first interpretation of various homographs, which can either be interpreted in a disorder-related or a disorder-unrelated manner (e.g., “port” can either be interpreted as a type of wine or as a harbor). In the domain of alcohol use, it has been shown that the extent to which participants interpret homographs in an alcohol-related manner predicted drinking behavior over and above demographic variables and explicit alcohol-related measures^22,23^. Extending the rationale of word association tasks, Woud and colleagues developed a novel paradigm in which they made use of more complex ambiguous cues, namely open-ended scenarios^19,20^ (for related paradigms see also^24–26^). As part of this, participants are provided with short scenarios, describing typical drinking situations. However, these scenarios do not have an ending and thus do not include a disambiguation related to the targeted behavior (i.e., “At the festival: You and your friends are attending a festival. You want to have a big night out. So, you and your friends are quickly going to the … “). Participants are asked to imagine themselves in the described scenario and then complete the scenario by writing down the first and spontaneous ending that comes to their mind (i.e., in the example scenario, possible continuations are e.g., going to the ‘bar’ or ‘stage’). The advantage of this approach is that such scenarios can target more complex and risk-prone situations compared to single cues. For instance, the specific content for alcohol-related scenarios in Woud et al.^19,27^ was created based on items of the Inventory of Drinking Situation (IDS;^28^), a well-validated questionnaire, which takes emotional, cognitive, and social antecedents of excessive drinking into account. Additionally, and unlike self-report measures that present participants with pre-defined statements or multiple-choice answering formats, the open-ended scenario approach allows for the assessment of idiosyncratic interpretations that are more representative of participants’ own thoughts^29^. Hence, the stimuli’s ecological validity is likely to be higher than in single-word cues. Adopting this approach, it has been demonstrated that heavy-drinking persons^19,30^ as well as alcohol-dependent inpatients^20^ generated more alcohol-related continuations than light-drinking individuals or control patients, being indicative of an alcohol-related interpretation bias. Furthermore, the tendency to endorse stronger alcohol-related interpretations in ambiguous situations predicted future drinking^27^. In addition, studies applying the open-ended scenario task to other areas of psychopathology have shown consistently that the number of disorder-related continuations was positively related to self-reported and/or observed levels of psychopathology. For example, Woud and colleagues found that a greater number of dysfunctional trauma-related appraisals was linked to more severe post-traumatic stress symptoms in an analog^29^ and inpatient sample^31^. In the context of sexual dysfunction, individuals with self-reported sexual difficulties rated ambiguous sexual scenarios more negatively and made more references to sexual problems^32^. Similar results were reported in the context of social anxiety^33^ and eating disorders^34^.

From a theoretical perspective, it is reasonable to expect that nicotine consumption or addiction is characterized by similar information processing (i.e., interpretation) biases as found in other substance-use and emotional disorders. For example, previous work has shown that people who smoke hold “compensatory health beliefs”, i.e., beliefs that one can compensate for the negative effects of smoking by engaging in healthy behaviors (^35,36^), which can in part explain continued tobacco consumption despite known adverse health effects. In addition, smoking-related implicit cognitions have been shown to play an important role in the maintenance of smoking behavior^3,13,37^. However, to the best of our knowledge, smoking-related interpretation biases have not been examined yet and thus remain to be shown in people who smoke. In addition, little is known as to whether the magnitude of such a cognitive bias varies in different groups, for instance as a function of behavior change (i.e., reduced consumption or achieved abstinence). This, however, is important when aiming to better understand the role of cognitive biases in the context of successful cessation versus relapse. Only few studies have compared implicit cognitive biases in individuals who smoke or those who ceased smoking. Regarding smoking-related approach and association biases, however, rather conflicting results have been reported^14,38^.

Accordingly, the objectives of the present study were two-fold. First, we aimed to transfer the rationale of examining smoking-relevant interpretation biases into the context of nicotine dependence, by using the open-ended ambiguous scenario approach and by testing different groups. Specifically, we tested people who smoke, people who had quit smoking, and people without a smoking history. Second, by including people who had stopped smoking and non-smoking individuals as additional groups, we aimed to better understand whether interpretation biases relate to different stages of substance use. Hence, our main objective was to enhance and update current perspectives on cognitive processes involved in nicotine addiction (i.e., dual process theories of addiction^1,2,4^), and to broaden our comprehension of factors related to abstinence. This, in turn, could provide new insights that may help to refine existing treatment approaches for nicotine dependence.

The integrative model for relapse situations and self-efficacy^39^ and the associated inventory (Self-Efficacy-Smoking; German version: SER-G;^40^) served as the foundation to create ambiguous scenarios relevant for cigarette smoking. The SER-G comprises various emotional and behavioral situations that can be attributed to positive/social, negative/affective, and habit/addictive situation types. Both the sum score and the scores for the specific situation types have been implicated in cigarette consumption and/or (re-)lapse situations^39^. Based on previous findings in the alcohol domain, we hypothesized that people who smoke, but not non-smoking individuals or people who had stopped smoking, generate more nicotine-relevant interpretations. Specifically, we had two expectations: First, we expected that smoking individuals generate more nicotine-relevant interpretations overall, i.e., across all types of smoking-relevant situations included in the scenario task. Second, we expected the same pattern when analyzing the different situations separately, such that smoking individuals generate more nicotine-relevant interpretations for each smoking-related situation type compared to the other two groups (e.g., people who stopped smoking and people without a smoking history). In addition, we expected the extent to which people who smoke complete the scenarios in a smoking-related manner to be positively related to daily cigarette consumption and the degree of nicotine dependence. Furthermore, results of a pilot study^41^ conducted by our working group using ambiguous open-ended scenarios based on the SER-G indicated that such scenarios were not only capable of tapping into smoking-related behavior but also triggered other substance-related endings (i.e., indications of alcohol and/or caffeine consumption) in non-smoking individuals. Hence, using the present data, additional exploratory analyses were conducted to investigate whether participants generated continuations that also included indications of other consumption behavior, and whether the extent to which they generated such continuations correlates with the corresponding behavioral counterparts. To do so, we included additional self-report measures assessing levels of alcohol and caffeine consumption.

## Results

### Participants’ characteristics

Participants were recruited from two study sites, the Ruhr-University Bochum (*n* = 102) and the University Siegen (*n* = 75). The total sample comprised *N* = 177 participants (active smoking individuals: *n* = 27; formerly smoking individuals: *n* = 30; never-smoking individuals: *n* = 120). Participants were on average 22.9 (*SD* = 5.6) years old. While 79% reported being female, 21% self-identified as being male. People who self-identified as active smokers smoked for 9.5 years (*SD* = 7.1), indicated to smoke an average of 9.4 (*SD* = 6.4) cigarettes per day, and had a FTND-score of 2.4 (*SD* = 2.5). According to the classification by Heatherton et al.^42^, those scores are indicative of a weak nicotine dependence. People who stopped smoking were in general abstinent for 3.2 (*SD* = 3.2) years and had smoked 4.8 (*SD* = 7.2) cigarettes per day prior to cessation. One hundred and thirty-nine (79%) participants indicated to consume alcohol, while the remaining participants stated to abstain from alcohol use. Of those consuming alcohol, the average AUDIT-C score was 2.8 (*SD* = 2.0), which does not indicate risky alcohol consumption (cut-off for risky consumption for females: > 3; for males > 4^43^). Regarding caffeine consumption, *n* = 99 (56%) participants indicated to drink at least one cup of coffee per day.

Awareness checks indicated that 103 (58%) participants were not aware about the study purposes. Forty-four (25%) participants had a vague idea (i.e., coping with stress-full life situations) and 30 (17%) participants had a more concrete idea about the study goals (i.e., investigating the relationship between being in every-day situations and consumption-related behavior).

### Smoking-related interpretation biases dependent on smoking status

**Figure 1:**
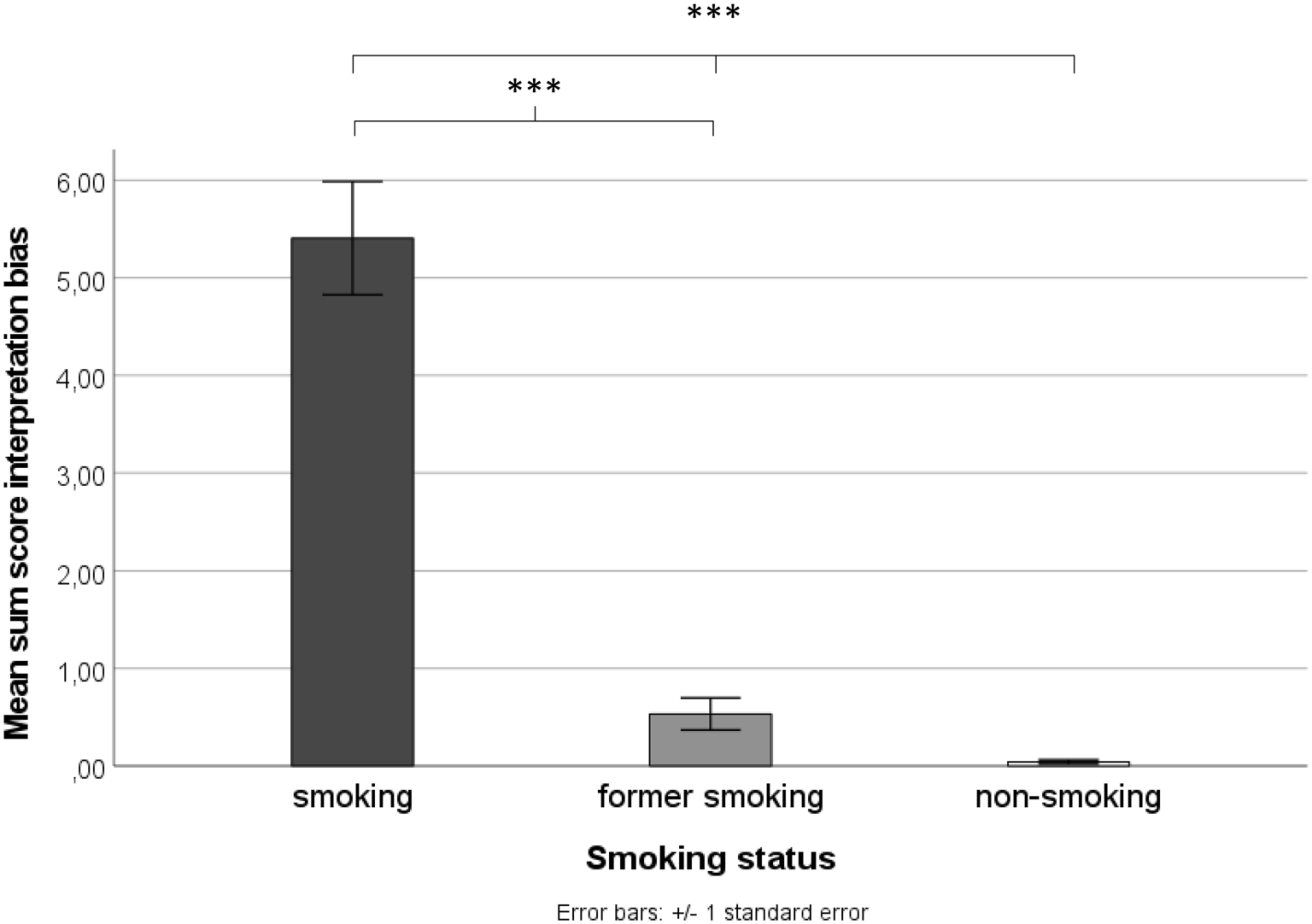
Overall interpretation biases for smoking-related situations as a function of smoking status. ***p < 0.001; **p < 0.01; *p < 0.05.

Figure 1 illustrates the magnitude of smoking-related interpretation biases for the three groups across all situation types (mean sum score) (please see the “Supplemental Material Appendix” [SMA], for illustrations based on study site). The univariate ANOVA revealed significant main effects for smoking status (*F*(2,171) = 200.05; *p* < 0.001; *ɳ*^*2*^ = 0.701) and study site (*F*(1,171) = 7.85; *p* = 0.006; *ɳ*^*2*^ = 0.044), and a significant two-way interaction between smoking status and study site (*F*(2,171) = 5.86; *p* = 0.003; *ɳ*^*2*^ = 0.064). Bonferroni corrected simple effect analyses revealed that across all three scenario types, active smoking individuals generated more smoking-related continuations than non-smoking individuals (*M*_*smoking individual*s_ = 5.24; *M*_*non-smoking individuals*_ = 0.04; *p* < 0.001) or people who had stopped smoking (*M*_*people who had stopped smoking*_ = 0.51; *p* < 0.001), being indicative of a smoking-related interpretation bias in the actively smoking group. No significant difference emerged between non-smoking individuals and people who stopped smoking (*p* = 0.160). Further, smoking individuals recruited from University Siegen produced more smoking-related continuations (*M*_*Siegen*_ = 6.13) than smoking individuals recruited from Ruhr-University Bochum (*M*_*Bochum*_ = 4.36; *p* < 0.001). However, the pattern of results (see above) was identical in both study sites (i.e., stronger smoking-related interpretation biases in people who smoke than former or non-smoking individuals).

**Figure 2:**
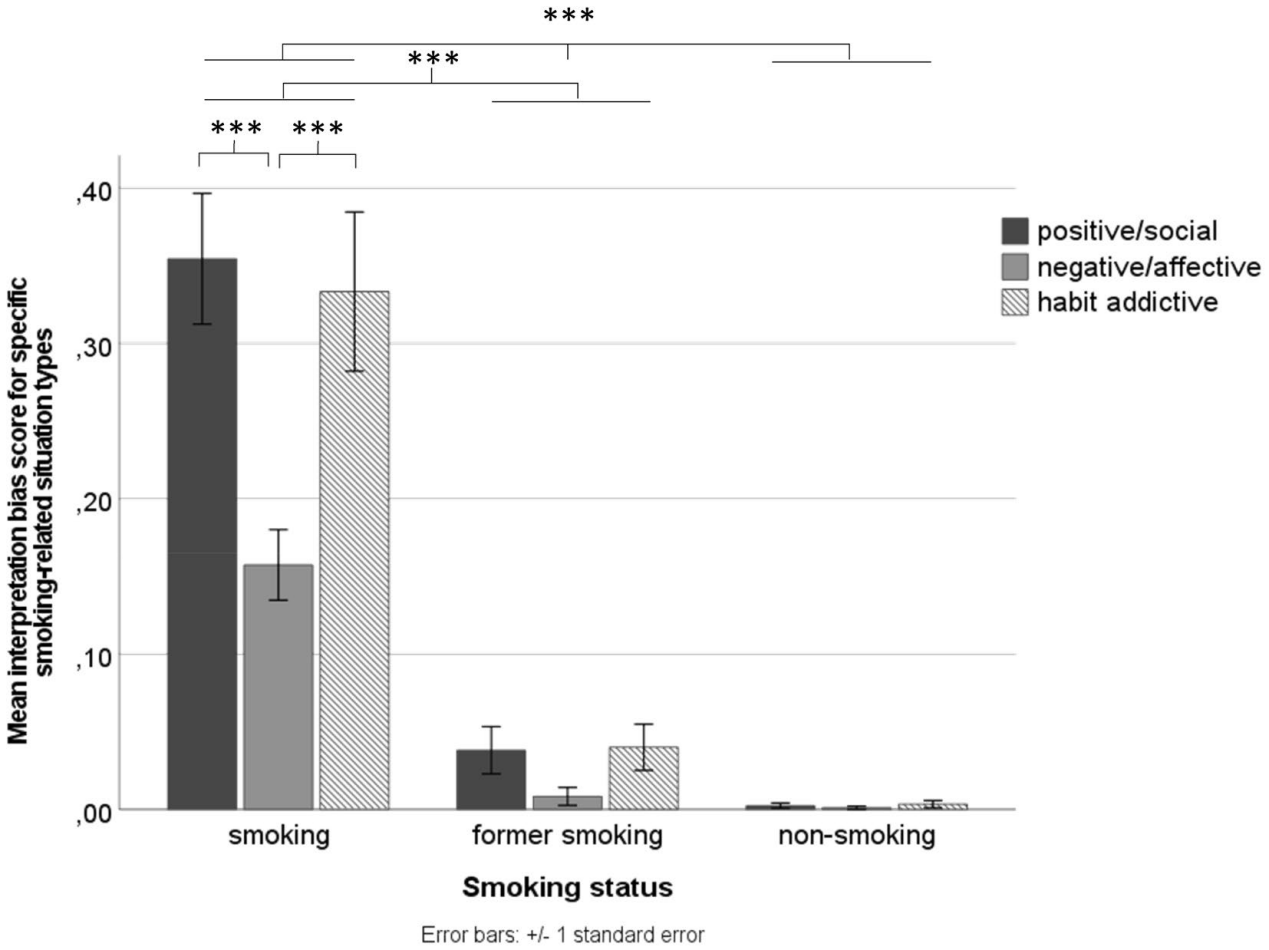
Interpretation biases for specific smoking-related situations as a function of smoking status. *** p < 0.001; **p < 0.01; *p < 0.05.

To explore the observed interpretation biases more thoroughly within the smoking-related scenarios, an additional mixed-design ANOVA was conducted, with differentiating between the three different smoking-relevant situation types (i.e., positive/social vs. negative/affective vs. habit/addictive) as the within-subjects variable and smoking status and study site as between-subjects variables (see Fig. 2). In line with the above findings, a significant main effect of situation type (*F*(2,170) = 65.10; *p* < 0.001; *ɳ*^*2*^ = 0.434), a significant main effect of smoking status (*F*(2,171) = 192.05; *p* < 0.001; *ɳ*^*2*^ = 0.692), and a significant two-way interaction between situation type and smoking status (*F*(4,342) = 27.84; *p* < 0.001;* ɳ*^*2*^ = 0.246) appeared. In addition, there was a significant main effect of study site (*F*(1,171) = 6.85; *p* = 0.010;* ɳ*^*2*^ = 0.039) and a significant three-way interaction between situation type, smoking status, and study site (*F*(4,342) = 3.87; *p* = 0.004;* ɳ*^*2*^ = 0.043). Bonferroni corrected simple effect analyses revealed that when separating for situation type, people who smoke produced more smoking-related continuations for positive/social, negative/affective, and habit/addictive situations than both non-smoking individuals (*ps* < 0.001) and people who had stopped smoking (*ps* < 0.001). The latter two groups (non-smoking individuals and people who had stopped smoking) did not significantly differ in terms of their interpretation biases for specific smoking-relevant situations (*ps* > 0.122). In addition, active smoking individuals were more likely to continuate positive/social (*M*_*positive/social*_ = 0.34) and habit/addictive situations (*M*_*habit/addictive*_ = 0.33) in a smoking-related manner than negative/affective ones (*M*_*negative/affective*_ = 0.15; *ps* < 0.001). When taking study site into account, analyses revealed that people who smoke recruited from University Siegen showed the strongest smoking-related interpretations for positive/social situations (*M*_*Siegen*_ = 0.42), followed by habit/addictive *(M*_*Siegen*_ = 0.34; *p* = 0.015), and negative-affective situations (*M*_*Siegen*_ = 0.19; *p* < 0.001). For people who smoke recruited from Ruhr-University Bochum, the magnitude of interpretation biases for positive/social (*M*_*Bochum*_ = 0.26) and habit/addictive situations (*M*_*Bochum*_ = 0.33) did not differ significantly (*p* = 0.161), but it was significantly greater than the one for negative/affective situations (*M*_*Bochum*_ = 0.11; *ps* < 0.001). Finally, smoking individuals from University Siegen exhibited significantly stronger interpretation biases in response to positive/social and negative/affective situations (*ps* < 0.001) than smoking individuals from Ruhr-University Bochum. No significant differences for study site appeared for habit/addictive situations (*p* = 0.106). Notwithstanding, smoking status remained a significant main effect factor, indicating that smoking individuals across both study sites produced more smoking-related interpretations than former- and non-smoking individuals (*ps* < 0.001).

Finally, to validate results by taking awareness about the study purposes into account, we calculated bivariate Pearson’s correlations between awareness levels (quantified as: 0 = *not aware*; 1 = *having a vague idea*; 2 = *aware*) and smoking-related interpretation biases. For neither group (*r*_*smoking individuals*_ = -0.006; *p* = 0.975; *r*_*people who had stopped smoking*_ = -0.013; *p* = 0.948; *r*_*non-smoking individuals*_ = -0.061; *p* = 0.507) significant correlations appeared, indicating no relationship between awareness and interpretation biases. In addition, we investigated task validity by performing additional analyses for the group of people who smoke. These analyses can be found in the SMA (A2).

### Correlations between smoking-related interpretation bias and nicotine consumption

Table 1 shows bivariate Pearson’s correlations between different interpretation bias indices (overall interpretation bias score across all scenarios and interpretation bias score per situation, i.e., positive/social, negative/affective, and habit/addictive) and smoking-related behaviors (cigarettes smoked daily, duration of use, and nicotine dependence as assessed with the FTND). Please note, that only smoking individuals were subjected to these correlational analyses (*n* = 27). For the overall interpretation bias, no significant correlations appeared with self-reported smoking behavior (*ps* > 0.165). However, there were significant positive correlations between interpretation biases for habit/addictive situations and cigarette consumption (*r* = 0.43; *p* = 0.029) and FTND-scores (*r* = 0.50; *p* = 0.008), indicating that the more smoking-related continuations in response to habit/addictive situations were generated, the higher the levels of daily tobacco use and the degree of nicotine dependence. The magnitude of significant correlations was moderate to high. Finally, there were no significant correlations between interpretation biases for habit/addictive situation types and duration of use (*p* > 0.779) or interpretation biases for positive/social or negative/affective situation types and smoking behavior (*ps* > 0.069).

**Table 1:** Pearson’s correlations (r) between interpretation biases for smoking-related situations and self-reported smoking-behavior in smoking individuals (n = 27).

Interpretation biases	Cigarette smoking behavior		
	Daily smoked cigarettes	Duration of use	FTND
Overall	0.09	− 0.18	0.28
Positive/social situations	− 0.26	− 0.16	− 0.11
Negative/affective situations	0.12	− 0.21	0.36
Habit/addictive situations	0.43**	− 0.06	0.50**

*p < 0.05, * p < 0.01, ***p < 0.001.

### Explorative analyses: Correlations between other consumption-related interpretation biases and consumption behavior

Table 2 shows bivariate Pearson’s correlations between alcohol- and caffeine-related interpretation biases and alcohol and caffeine consumption. Please note that in four cases, endings were entered, which related to two different substances (i.e., a nicotine- as well as an alcohol-related ending). In line with the predefined coding approach, the first entered substance was coded. However, results remained the same when both or none of the two substances were coded. Only participants who indicated to consume alcohol (*n* = 139) or coffee (*n* = 99) were subjected to these additional analyses. Significant positive correlations emerged between alcohol-related continuations for positive/social (*r* = 0.36; *p* < 0.001), negative/affective situations (*r* = 0.25; *p* < 0.003) as well as the sum score for all three situation types (*r* = 0.26; *p* < 0.002) and the intensity of alcohol use as indicated by the AUDIT-C scores, respectively. Hence, the tendency to interpret ambiguous (particularly positive/social and negative/affective) situations in an alcohol-related manner was positively associated with the extend of alcohol consumption. For coffee-drinking persons, positive correlations between caffeine-related continuations for all three ambiguous scenario types and the number of cups of coffee drunk per day emerged (*rs* ≥ 0.21; *ps* < 0.05).

**Table 2:** Pearson’s correlations (r) between interpretation biases for consumption-related situation and self-reported consumption behavior.

Interpretation biases	Other consumption behavior	
	Alcohol use (AUDIT-C)	Caffeine use
	n = 139	n = 99
Positive/social situations	0.36***	0.25*
Negative/affective situations	0.25**	0.23*
Habit/addictive situations	0.07	0.21*
Sum score	0.26**	0.28**

*p < 0.05, **p < 0.01, ***p < 0.001.

## Discussion

The present study is the first to investigate smoking-related interpretation biases in a group of active smoking individuals, people who had stopped smoking, and non-smoking individuals. To do so, a novel open-ended scenario task was implemented, which was based on preliminary work in the field of alcohol (mis-)use^19,20,25,44^ and other emotional disorders^29,32,33^. Our results indicated that active cigarette-smoking individuals were more likely to endorse a smoking-related interpretation when faced with ambiguous, potential smoking-related situations than former- or non-smoking individuals. In particular, more smoking-related continuations were generated in response to positive/social and habit/addictive situation types as compared to negative/affective ones. Moreover, within the group of smoking individuals, analyses revealed a positive association between the tendency to interpret habit/addictive situations in a smoking related manner and daily nicotine consumption as well as levels of nicotine dependence. This is in line with results from previous studies using the scenario task, revealing a close relationship between disorder-specific continuations generated on the scenario task and levels of psychopathology^30–32^. Our findings can be interpretated in the context of memory association research and schema formation (for a similar reasoning, see^20,21^. That is, it is likely that while reading and imagining ambiguous, smoking-related scenarios, smoking-related memory associations became activated in smoking individuals, which were developed and reinforced through past experiences and current behavior in similar real-life situations (i.e., actualization of events, in which participants reached for a cigarette). As such, smoking-related continuations may have become more accessible and easier to (re-)produce. The fact that people who had stopped smoking also generated smoking-related continuations but fewer than individuals who actively smoked may indicate that smoking-related memory associations still exist in that group but are weaker in terms of their activation and/or inhibited by other structures. Finally, most non-smoking individuals did not produce any smoking-related continuations. Taken together, this could imply that the presence of smoking-related interpretation biases can not only be explained by means of (past) cue exposure but should be regarded as a dynamic process that is capable of change.

However, within the group of actively smoking individuals, we did not find significant correlations between interpretation biases across all smoking-related scenarios (sum score) or interpretation biases in response to positive/social and negative/affective situation types and self-reported smoking behavior (assessed via the FTND and cigarette consumption). Both measures of smoking-behavior are strong correlates of nicotine addiction—hence, significant relationships between these behaviors and interpretation biases were expected. However, maybe other smoking-related indices would have been better measures in this context, and a better match in terms of assessed concepts, e.g., subjective attitudes towards smoking operationalized via the dimensions liking/wanting (see e.g.,^45^. In addition, it is conceivable that other factors such as smoking deprivation duration or motivation to cease smoking could have influenced (i.e., hampered) the process of generating smoking-related interpretation biases. In fact, research suggests that nicotine deprivation may affect approach associations towards smoking cues^14^ and that motivational aspects can influence the magnitude of cognitive biases^46^. Another explanation for our finding is related to statistical power: Our a priori power analysis was based on finding an at least moderate-sized effect. It might be that smaller, yet still relevant correlations exist, however, such correlations can only be detected in larger samples. Generally, it should be noted that cigarette smoking is a highly automated type of behavior as evidenced by extremely high percentage rates of nicotine dependence even in relatively light-smoking individuals^47^. This might have been reflected in our finding of associations between self-report data and interpretation biases for habit/addictive situations, which are rather independent from specific mood states (positive as well as negative ones).

In addition, we detected a difference in the magnitude of smoking-related interpretation biases between study sites, with people who smoke recruited from University Siegen generating more smoking-related continuations than those recruited from Ruhr-University Bochum. This was an unexpected finding since both universities are geographically close, and no demographic differences in the respective subsamples appeared. It may be that unforeseen time-dependent effects (i.e., different examination phases in the respective universities, which may have led to different levels of stress) were involved here. However, the general pattern of results is comparable across both sites, such that smoking individuals from both universities were characterized by stronger smoking-related interpretation biases than former- or non-smoking individuals. In addition, due to small sample sizes per group, the reported differences between study sites could have been spurious and thus should be interpretated with caution.

A secondary outcome in this study was that exploratory analyses revealed correlations between other alcohol- and caffeine-related interpretations in response to the ambiguous scenarios and self-reported alcohol and caffeine consumption. Although smoking-relevant scenarios were created based on the SER-G^40^, a well-validated self-report measure that comprises different types of high-risk smoking situations, it may be that the included situations elicited certain emotions, moods, or habits, which in turn lead to automatic consumption or substance-based interpretations beyond smoking-related interpretations. For instance, it appears that participants tended to automatically associate emotional situations (i.e., feeling stressed) with consuming substances that are known to influence one’s mood. These findings provide further support for the applicability of the present scenario-based task, which is not only capable of capturing interpretation biases associated with nicotine-related behavior but, when expanded to other substance-use behaviors or to other groups of participants, is also sensitive to measuring associated interpretation biases. However, these results also clearly reduce the task’s internal validity, meaning that the current task taps into a more general interpretational process, namely the tendency to respond to emotion-eliciting events in an externalizing (consumption-related) manner. However, additional analyses that can be found in the SMA (A2) provide evidence that the present open-ended scenario task is indeed more specific to capture smoking-relevant interpretations since people who smoke produced significantly more smoking- than alcohol- or caffeine-related interpretations. That said, future studies should explore the feasibility and merit of creating more fine-grained smoking-relevant scenarios that activate interpretations unique to cigarette smoking, thereby increasing the internal and discriminative validity of the task and explain additional variance in smoking behavior.

Next to notable strength of the study, some limiting factors must be considered. First, the present study was designed as a first investigation of nicotine-related interpretation biases. As such, the sample size comprising smoking individuals and people who had stopped smoking was rather low, although in accordance with the power analysis. Hence, replication in a larger sample would be desirable. Another related issue concerns the sample’s characteristics. Recruited participants were mostly young students and those who smoked reported a relatively short history of smoking or levels of nicotine dependence. Hence, generalization of study results might be limited when considering other groups of smoking individuals, especially older or severely dependent persons. Also, the verbal nature of the task requires certain skills for it to be performed, for instance imagery, introspection, and familiarity with expressive writing. It is therefore uncertain whether this can be transferred to other populations that are less familiar with these requirements. Finally, given the fact that our sample comprised college students, scenarios depicted typical students’ situations. Thus, transferring the task to samples other than students would require an adaptation of the specific scenarios (see SAM, A3, for the precise contents of the scenarios). Further, although our scenario task can be thought of as an indirect approach as participants’ interpretations are inferred from their performance on the task (as opposed to a direct inquiry;^13^), it remains uncertain as to which extent this task taps into implicit information processing or whether measures derived from the task are confounded by reflective reasoning. For instance, other than in reaction time-based paradigms, participants had time to reflect on their answers and/or could have revised initial responses. However, awareness did not correlate with interpretation biases, and we strived to use instructions which favor the activation of implicit processes (i.e., by instructing participants to provide their first, most spontaneous response). Moreover, the distinction between deliberate versus implicit processes may be useful from a theoretical point of view (but see^4,5^ for a critical discussion on dual process accounts); however, some authors note that the underlying processes cannot be strictly matched to a distinct category (i.e., reflective vs. automatic) and argue for a continuum-based approach. This means that in the process of response selection, different degrees of reflectivity and/or automaticity are likely (for a similar reasoning, see^48^. Hence, future research should combine the scenario task with other more direct (i.e., self-report) and indirect (i.e., reaction time-based tasks) measures to advance our understanding of the different processing levels. For example, smoking-relevant associations could be assessed via different IAT versions, and alternative versions of the scenario task could be used to infer interpretation biases (e.g., by recording categorization times of probes that disambiguate open-ended, ambiguous scenarios in a smoking-related or unrelated manner). In doing so, reaction times would serve as additional indices of interpretation biases and could therefore cross-validate them. In addition, future studies using the open-ended scenario task could also add a time-limit to the response format, thereby inciting more automatic, harder to control continuations. Finally, an alternative explanation that could account for the observed results is that the generated continuations do not only reflect an interpretation bias but are the product of a response bias. Specifically, the generated continuations may be reflective of how participants responded to similar situations in the past, without representing an interpretational component in the here and now. Such an explanation, however, poses a threat to the task’s internal validity and thus is an important target for future studies.

Our work has clear implications for both basic clinical research and interventions. For instance, if replicated, the current open-ended scenario task may have potential to be used in therapy (next to other self-report and clinical measures) as it might provide unique insights into the patient’s ideographic interpretation styles in various emotion-activating or habitual situations. Specifically, it could be used to identify individual high-risk situations, which can then become a specific target in psychotherapy (i.e., for stimulus control, planned cue exposure, or as part of relapse prevention). Besides, the open-ended scenario task might provide an enhanced understanding of driving factors that contribute to dysfunctional behavioral patterns over and above traditional self-report and may therefore be used as a treatment target itself, for example as a starting point for cognitive restructuring^49^. Indeed, some evidence exists for the notion that encouraging people who smoke to regulate emotions via deliberate reappraisal has beneficial effects on cigarette craving^50^.

The finding that people who had stopped smoking showed weaker smoking-related interpretation biases suggests that such biases may be subject to change, and that their appearance may depend on whether a person actively engages in smoking behavior or not. Certainly, longitudinal research is needed to answer the question as to whether a substance-related interpretation bias is an antecedent of addiction and/or a consequence, and which role it plays in the context of cessation and of course relapse. An interesting question for further research would be to determine whether a weaker bias is associated with successful abstinence and/or whether bias retention predicts relapses. With regards to the current sample, it is noteworthy that as small proportion of people who had stopped smoking generated some smoking-related continuations (see Fig. 1). Hence, longitudinal research is needed to investigate whether such individuals are at higher risk of relapse.

In a related vein, the last decade has witnessed a steady development of a new line of research, summarized as Cognitive Bias Modification (CBM;^51^). CBM is based on the assumption that cognitive biases causally contribute to maladaptive emotional states and/or behaviors^1^. This, in turn, motivated researchers to test whether modifying cognitive biases (i.e., reduce threat-related interpretations, induce benign interpretations or positive biases) in clinical samples may have and/or improve therapeutic effects^52^. To do so, different training paradigms were developed (for a review in the context of consumption behavior, see^53^). For instance, in the context of CBM for interpretation (CBM-I), Woud et al.^26^ conducted an experimental proof-of-principle study and trained heavy-drinking individuals to resolve ambiguous alcohol-related scenarios in an alcohol-related or a neutral manner, showing that participants in the alcohol training group interpreted ambiguous scenarios as more alcohol-related after training. However, there was no reduction in alcohol-related interpretation biases in the neutral training group and the training did not affect drinking behavior, which was assessed via a bogus taste test. A comparable study conducted by Salemink et al.^44^ reported similar findings, showing that a CBM-I training designed to modify alcohol-related interpretation biases in negative-affect drinking was successful in reducing such biases but failed to provide evidence for affecting drinking behavior. Hence, more research is needed to expand our understanding of the potential causal role of interpretation biases and how this transfers to behavioral outcomes. This can be done via experimental research as well as appropriately-designed clinical studies. As of yet, such procedures, i.e., training procedures to modify interpretation biases, are still lacking for nicotine addiction. However, a recent meta-analysis by Martinelli et al.^54^ provided support for the notion that CBM-I has merit in the treatment of various mental disorders, including substance use and eating disorders. Besides, CBM approaches to retrain other cognitive bases (i.e., automatic approach tendencies) have yielded some promising results with regards to reduced nicotine consumption^16,55^ or increased abstinence rates^56^ (but see^57^, who did not report a training-specific effect). Hence, providing CBM-I to e.g., smoking individuals who are motivated to cease could constitute a promising new avenue for future research and may have potential to complement existing conventional cessation interventions.

Taken together, the present study is the first to demonstrate a smoking-related interpretation bias in people who smoke compared to former- and non-smoking individuals by means of a novel open-ended scenario approach. By doing so, this study took the first step in a multistage translational process towards further investigating biased interpretational processes in the context of nicotine dependence and the development of potential computerized trainings^58^. Prior to implementing clinical translations, however, verification of effective target engagement and its testing by means of randomized controlled trials, the identification of a reliable cognitive target, and its association with specific dysfunctional behavior or symptoms is a vital prerequisite. Further research is needed to elucidate in how far such biased information processing patterns contribute to the initiation, maintenance, and recovery from psychological disorders, including nicotine addiction. Notwithstanding, the present study demonstrated that smoking-related interpretation biases are present in active smoking individuals, but not in people who had stopped smoking, indicating that such biases may fluctuate. In addition, our findings replicated and extended previous results on the relationship between alcohol-related interpretation biases and alcohol consumption as well as other consumption-related behavior (i.e., caffeine consumption). An avenue for future research would be to examine whether the present scenario-based paradigm is sensitive to change and whether the task could also be modified into a training variant to aid interpretation bias re-training for smoking.

## Methods

### Power analysis

A total sample size of *N* = 159 (i.e., *n* = 53 per group) was determined via an a priori power analysis (G*Power 3.1;^59^): We aimed to achieve 80% power to detect a between-group effect of *d* = 0.50 at *p* < 0.05 for a three-group design on our outcome of main interest (e.g., that people who smoke generate more nicotine-relevant interpretations overall and within specific smoking-related situation types). For correlational analyses (e.g., correlations between interpretation biases and self-reported consumption behavior), at least 29 participants were required for a correlation of *r* > 0.50, a power of 0.80 and a two-tailed significance test at *p* < 0.05. Effect sizes were expected to be moderate, based on previous research^19,20^.

### Participants

Data collection took place at two study sites, at Ruhr-University Bochum and University Siegen. Recruitment was accomplished via flyers and advertisement in lectures. Interested individuals were provided access to an online survey by means of a study link (provided via the Internet platform LimeSurvey). Exclusion criteria were a history of major medical or psychiatric disorders or insufficient German language skills. A total of *N* = 218 students started the survey (Ruhr-University Bochum site: *n* = 121; University of Siegen site: *n* = 97). However, not all participants completed the scenario task or did not provide the required data on the self-report measures. After exclusion of these participants, the final sample for the present analyses was* N* = 177. Of these, *n* = 27 indicated to be regular, active smoking individuals, while *n* = 30 indicated to have stopped smoking. The remaining participants did not have a history of smoking (*n* = 120). Participants received course credit (1/2 h) for participation. Participation was voluntary and participants had the right terminate the survey or to withdraw their consent for participation at any time.

### Procedure

To avoid expectancy or priming effects, the study was described as an investigation of leisure behavior and coping with different situations. Therefore, participants were not screened for (nicotine) consumption behavior prior to study inclusion. After participants provided informed consent, demographic questionnaires were applied. Thereafter, participants were presented with the open-ended scenario task and were instructed to enter their first and spontaneous continuation as free text as quickly as possible. After completing the scenario task, questions on consumption-behavior (i.e., nicotine, alcohol, and caffeine consumption) were presented. To further distract participants from the study goal, additional filler questions (i.e., questions about hobbies, pets) were administered, which were neither part of the research questions nor final analyses. Finally, participants were provided the opportunity to indicate their ideas about the study’s aim (i.e., assessed via the following item: “Lastly, we are interested in your ideas about what this study might be about. Please enter your thoughts, if applicable”).

### Measures

#### Open-ended scenarios

Interpretation biases were assessed by means of our newly developed open-ended scenario task that aimed to tap into interpretational processing biases related to cigarette smoking. Each scenario described typical student life situations and started with a title that was followed by three lines of text. The final line ended abruptly. Participants were instructed to imagine themselves in the described situation and to enter the first continuation that came to mind. The scenario task comprised 20 smoking-related and 20 neutral situations. Task instructions as well as the exact scenarios (both in the original German version as well with English translations) are presented in the SMA (A3). Prior to this study, each scenario was extensively piloted to check for comprehensibility and readability, and to ensure that various idiosyncratic interpretations were possible (i.e., that ambiguous smoking-related situations could also be interpreted in a non-smoking manner)^41^. The integrative model for relapse situations and self-efficacy^39^ and the associated inventory (SER-G;^40^) served as the foundation to create smoking-related situations. More specifically, the SER-G comprises three broad types of high-risk situations, which are capable of triggering smoking behavior and leading to (re)lapses: Positive/social situations, negative/affective situations, and habit/addictive situations. For example, the factor “positive/social” was operationalized by means of the following scenario: *“It's a lovely day and you are lying next to a beautiful lake. While you are relaxing, you think there's just one thing missing. So you reach into your bag and ……”*. The following scenario serves as an example for the factor “negative/affective”: *“Your roommate makes a huge mess in your apartment. When you confront them, you and they end up in an argument. To clear your head, you leave the apartment and get yourself a …”*. Finally, an example for the “habit/addictive” factor is: *“You want to take the bus into town. Today, of all days, the bus is running late. While you stand at the bus stop and wait, you pass the time with …”*. In line with the SER-G, seven positive/social, eight negative/affective and five habit/addictive situations were created. Internal consistency for the herein created open-ended scenario task was good for the full scale (Cronbach’s *α* = 0.844).

**Figure 3:**
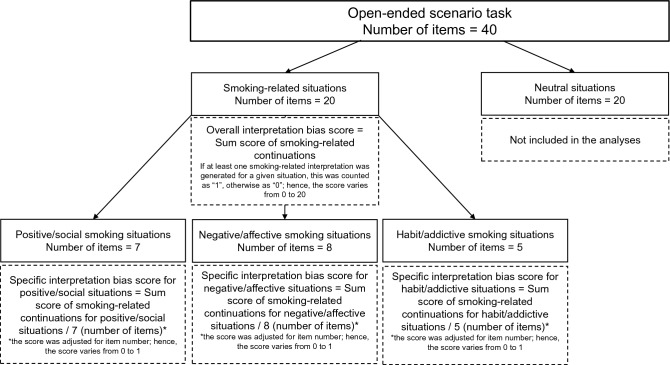
Open-ended scenario task and interpretation bias score calculation.

Neutral situations were based on Woud et al.^19^ and were further expanded (example: “*Every other weekend you play 'Risk' with your friends. Everything is set up and the missions are distributed. This time your mission is very …*”). Neutral scenarios served as control and filler situations and were not included in the main analyses. Reasons for inclusion were to confirm the cover story that the task was designed to assess students’ leisure behavior and to help keeping participants being unaware of the study’s purposes. Due to the design, content, and nature of the scenarios (i.e., grammatical composition at the end of the sentence), we did not expect any addiction-related continuations here (but see^19^ for generalization effects to neutral scenarios). A detailed illustration of the task and different scenario types is presented in Fig. 3.

#### Smoking-related measures

All participants were asked to indicate their current and past smoking status. Smoking individuals indicated the intensity (daily smoked cigarettes) and duration (in years) of use and filled out the Fagerström Test for Nicotine Dependence (FTND;^42^; German version:^60^). The FTND consists of six items capturing the degree of nicotine dependence (i.e., “*How soon after you wake up do you smoke your first cigarette?*”) on a range from 0 to 10. In this sample, internal consistency was acceptable (Cronbach’s *α* = 0.752). People who had stopped smoking indicated the duration of abstinence (in years) and the amount of daily smoked cigarettes before abstinence.

#### Alcohol-related measures

All participants filled in the Alcohol Use Disorders Identification Test alcohol consumption questions (AUDIT-C) as a brief screening test for heavy drinking and/or active alcohol abuse or dependence^61^. The AUDIT-C can be considered an efficient and valid short version of the AUDIT^43^ and comprises the first three items of the AUDIT (i.e., alcohol use: i) “*How often did you have a drink containing alcohol in the past year?*”; drinking intensity: ii) ”*How many drinks did you have on a typical day when you were drinking in the past year?* ”; iii) ”*How often did you have 6 or more drinks on one occasion in the past year?*”). The AUDIT-C adopted the same response options as the AUDIT with sum scores ranging between 0 and 12. In this sample, internal consistency was rather low (Cronbach’s *α* = 0.650).

#### Caffeine consumption

To assess caffeine consumption, participants were asked whether they consume coffee. If answered with yes, participants had to indicate their daily coffee consumption on a single-item Likert-scale (0 = 0 cups of coffee/day; 1 = one cup of coffee/day; 2 = two cups of coffee/day; 3 = three cups of coffee/day; 4 =  > three cups of coffee/day).

### Independent coding of scenario continuations and interpretation bias calculation

Two trained psychologists blind to participants’ smoking status rated the generated continuations in response to both smoking-related and control scenarios in a conservative manner. That is, only unambiguous smoking-related continuations were rated. In case multiple smoking-related endings were generated as a continuation of a single scenario, these endings were counted only once. In addition, as our pilot study suggested that the ambiguous smoking-related scenarios were also capable of eliciting more general consumption-related continuations, alcohol- and caffeine-related continuations were also included in the ratings to conduct explorative analyses. If participants generated endings related to different substances (i.e., a nicotine- as well as an alcohol-related ending), the first entered substance was coded as we intended to tap into the most spontaneous continuation. Inter-rater reliability for the coding of ambiguous smoking-related continuations was almost perfect (Cohen’s *κ* for positive/social situations = 0.977; Cohen’s *κ* for negative/affective situations = 0.954; Cohen’s *κ* for habit/addictive situations = 0.905; Cohen’s *κ* across all smoking-relevant situations = 0.947; *ps* < 0.001). Both raters coded the scenarios independently and ratings were compared for concordance afterwards. In case of disagreement, raters discussed on the critical coding and agreed on a consensus. As expected, the independent raters did not report any smoking-related continuations for neutral scenarios, implying a 100% agreement and confirming that these scenarios served as filler.

An overall smoking-related interpretation bias was derived from the sum score of continuing ambiguous smoking-relevant scenarios in a smoking-related manner. Therefore, the score could vary between 0 and 20, as the total number of smoking-relevant situations was *n* = 20. As mentioned earlier, (see “Open-ended scenarios” in the Measures subsection), these 20 smoking-relevant situations can be divided into 3 specific high-risk smoking situations: Positive/social (*n* = 7), negative/affective (*n* = 8), and habit/addictive *(n* = 5) situations. This specification was made to investigate whether the activation of smoking-related interpretation biases would depend on the specific type of smoking-related situation presented. For reasons of better comparability and because the specific situation types differed in their numbers of items, we created mean scores for each specific situation type (i.e., sum score divided by total number of items per situation type). Hence, the smoking-related interpretation bias scores could vary between 0 and 1. Figure 3 provides a detailed illustration of the open-ended scenario task as well as interpretation bias scores derived from the task.

### Data preparation and statistical analyses

Data were analyzed using IBM SPSS Statistics for Windows, version 27^62^. To explore whether people who smoke generate more smoking-related continuations across all smoking-related, open-ended scenarios, than non- or former smoking individuals, a univariate ANOVA with the between-subjects factors “smoking status” and “study site” was conducted. Eta squared was used as a measure for the effect size. To further explore whether the bias’s extent depends on specific smoking-related situation types, a mixed design ANOVA was conducted with “smoking status” and “study site” as between-subjects factors and smoking-related interpretations for i) positive/social, ii) negative/affective, and iii) habit/addictive smoking-relevant situations as within-subjects factor. To account for the Type II error rate, a multivariate approach was applied as it takes possible correlations between dependent variables into account. In addition, to control for the Type I error rate, follow-up analyses were conducted using the Bonferroni correction.

Bivariate Pearson’s correlations were calculated in the group of actively smoking individuals to test whether smoking-related interpretation biases (across all scenario types and for the specific smoking situation types) were positively related to the extent of nicotine consumption and the degree of nicotine dependence. For the explorative analyses, which examined whether participants generate other consumption-related continuations in response to ambiguous smoking-relevant situations and whether this interpretational pattern is related to the corresponding behavior, bivariate Pearson’s correlations were calculated as well.

### Ethics approval and consent to participate

The study protocol was approved by the local Ethics Committee of Ruhr-University Bochum (ethical approval number: 20110512) and the University Siegen (ethical approval number: LS_ER_77) and was conducted in accordance with the Declaration of Helsinki and Good Clinical Practice guidelines. The authors have complied with APA ethical standards in the treatment of their sample.

### Supplementary Information

Supplementary Information.

## Data Availability

The dataset used and analyzed during the current study as well as study materials are available at https://osf.io/muz25/?view_only=0b72b1210ff2435c89fb14d31b46b803.
