# Supplementary material for: Nicotine-related interpretation biases in cigarette smoking individuals
Source: Sci Rep. 2024 Feb 27;14:4796. doi: 10.1038/s41598-024-55256-6 (PMC10899185; doi:10.1038/s41598-024-55256-6)
Supplement: Supplementary file 1 — Supplementary Information. [file 41598_2024_55256_MOESM1_ESM.pdf]

Supplemental Material Appendix

Here, we present additional analyses based on study site (A1) and a more in-depth investigation of the validity of our newly developed open-ended scenario approach (A2). In addition, instructions for participants and scenarios used for the open-ended scenario task are presented (A3).

A1: Additional analyses based on study site

Smoking-related interpretation biases dependent on smoking status and study site

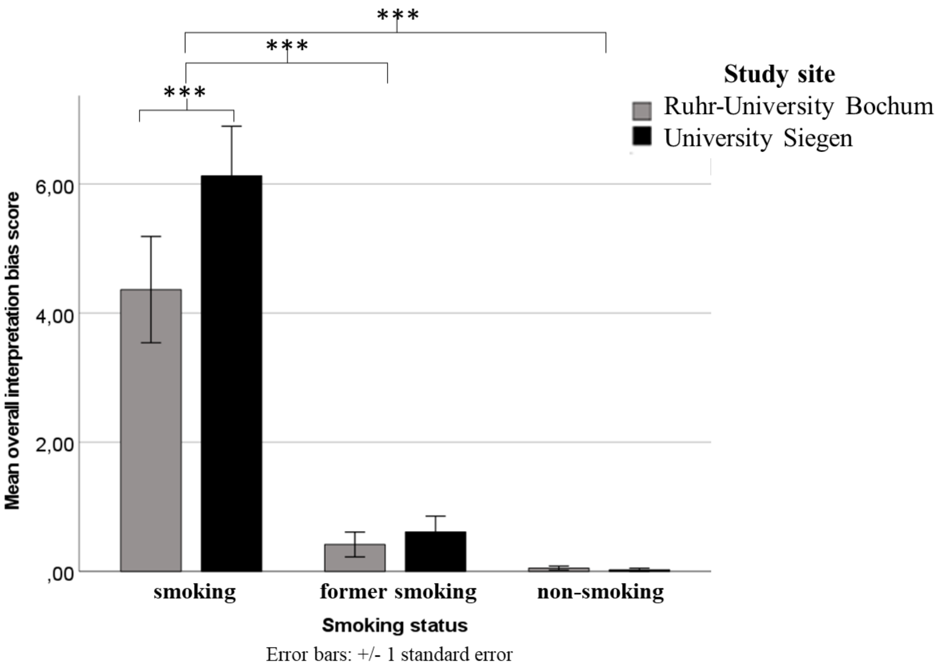

Figure A1(a). Overall interpretation biases for smoking-related situations as a function of smoking status and study site. Note. *p < .05, **p < .01, ***p < .001

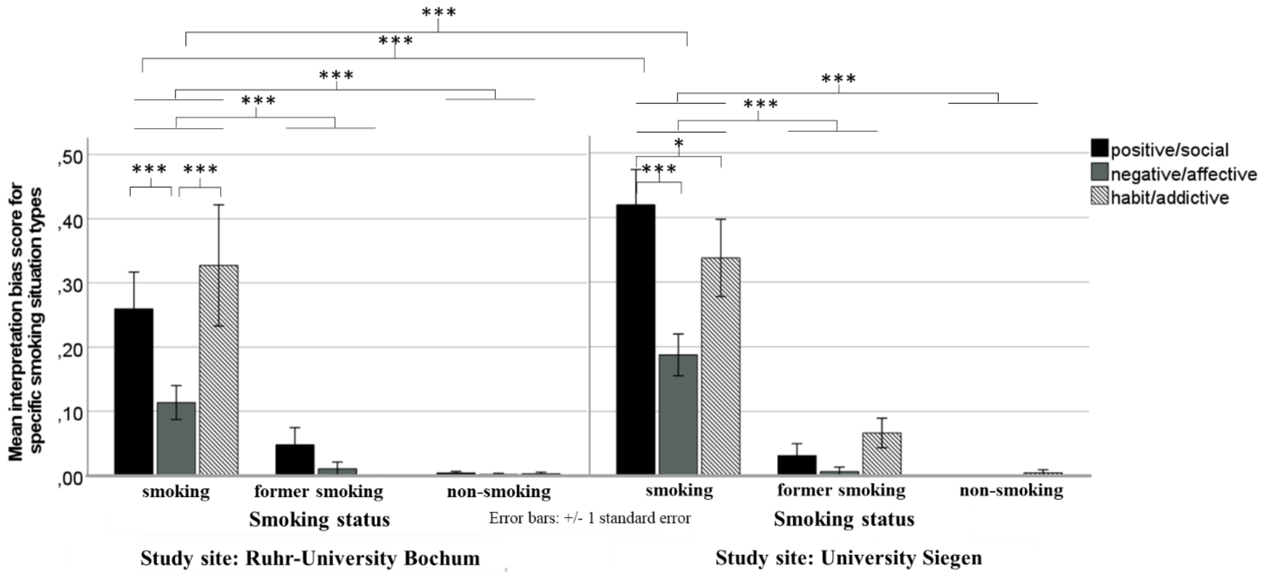

Figure A1(b). Interpretation biases for specific smoking-related situations as a function of smoking status and study site. Note. *p < .05, **p < .01, ***p < .001

A2: Additional investigation of task validity

To investigate the validity of our newly developed smoking-relevant open-ended scenario approach in more detail, we performed additional analyses for the group of people who smoke. More precisely, paired t-tests were conducted to determine whether people who smoke would generate more smoking-related than other substance-related (i.e., alcohol or caffeine) continuations in response to smoking situations, providing additional support for the task's internal validity.

In line with our reasoning, results indicated that people who smoke produced more smoking- than alcohol-related continuations across all smoking-relevant scenarios ($M_{smoking\_continuations} = 5.41$; $M_{alcohol\_continuation} = 1.07$; $t(26) = 6.36$; $p < .001$; standardized Cohen's $d = 3.54$). In addition, people who smoke produced more smoking- than caffeine-related continuations across all smoking-relevant scenarios ($M_{caffeine\_continuation} = .93$; $t(26) = 7.38$; $p < .001$; standardized Cohen's $d = 3.15$). The same was true for interpretation biases for specific smoking-relevant situation types (comparisons with alcohol-related continuations: positive/social: $M_{smoking\_continuations} = 2.48$; $M_{alcohol\_continuation} = .96$; $t(26) = 3.93$; $p < .001$; standardized Cohen's $d = 2.01$; negative/affective: $M_{smoking\_continuations} = 1.26$; $M_{alcohol\_continuation} = .41$; $t(26) = 3.69$; $p < .001$; standardized Cohen's $d = 2.04$; habit/addictive: $M_{smoking\_continuations} = 1.67$; $M_{alcohol\_continuation} = .11$; t(26) = 5.79; $p < .001$; standardized Cohen's $d = 1.40$; comparisons with caffeine-related continuations: positive/social: $M_{caffeine\_continuation} = .18$; $t(26) = 7.29$; $p < .001$; standardized Cohen's $d = 1.64$; negative/affective: $\mathrm{M}_{\mathrm{caffeine\_continuation}} = .78$; t(26) = 2.23; $p = .035$; standardized Cohen's $d = 1.12$; habit/addictive: $M_{caffeine\_continuation} = .30$; $t(26) = 5.23$; $p < .001$; standardized Cohen's $d = 1.36$).

Hence, these additional analyses provide evidence that the present open-ended scenario task is more specific to capture smoking-relevant interpretations in people who smoke instead of eliciting general substance- or consumption-related interpretations.

A3: Detailed task instructions and scenarios used for the Open-ended ambiguous scenario task for smoking

Task instructions

German Version:

Herzlich willkommen zu dieser Aufgabe!

Auf den folgenden Seiten werden Sie 40 kurze Geschichten finden. Sie alle beschreiben Situationen aus dem Alltag eines Studenten.

Sie werden sehen, dass die Geschichten noch kein Ende haben. Ihre Aufgabe ist es, jede Geschichte zu beenden, indem Sie den letzten Satz vervollständigen.

Während der Vervollständigung der Sätze, ist Folgendes wichtig:

- Versuchen Sie, sich selbst in den Situationen vorzustellen, auch wenn diese nicht perfekt mit Ihren alltäglichen Situationen zusammenpassen.
- Lassen Sie sich von Ihrem ersten spontanen Eindruck leiten. Denken Sie nicht zu lange nach; Schreiben Sie einfach auf, was Ihnen als erstes in den Sinn kommt.

Es gibt keine richtigen oder falschen Antworten. Die Bearbeitung dieser Aufgabe wird ca. 10 Minuten in Anspruch nehmen.

English Translation:

Welcome to this task!

On the following pages, you will find 40 short stories. All stories describe situations from the everyday life of a student.

You will see that the stories are without an ending yet. Your task is to finish each story by completing the last sentence.

While completing the sentences, the following is important to keep in mind:

- Try to imagine yourself in the situations, even if they do not match with your everyday situations perfectly.
- Let your first spontaneous impression guide you. Do not think too long; just write down the first thing that comes to your mind.

There are no right or wrong answers. This task will take about 10 minutes to complete.

Smoking-relevant scenarios:

Please note that the order of scenarios were randomized for participants.

German Version:

Scenario type/Title:	Scenario
**Positive/social**	
Ein Tag am See	Es ist ein herrlicher Tag und du liegst an einem schönen See. Während du entspannst, denkst du, dass nur noch eine Sache fehlt. Also greifst du in deine Tasche und …
Bier trinken	Du bist mit Bekannten in einer Gaststätte. Ihr unterhaltet euch und trinkt ein paar Flaschen Bier. Du merkst, beim Trinken steigt dein Verlangen nach …
Vor der Disco	Du wartest mit ein paar Freunden in der Schlange vor einer Disco. Einer von ihnen dreht sich zu dir und fragt „Willst du auch?“. Du bejahst und nimmst dir …
Auf dem Weg zur Party	Du bist auf dem Weg zu einer Party. Auf dem Weg dorthin merkst du, dass du etwas vergessen hast. Also läufst du zum nächsten Kiosk und kaufst …
Kaffee trinken	Du triffst dich mit einer Freundin, die du schon länger nicht mehr gesehen hast. Während ihr euch unterhaltet, trinkt ihr eine Tasse Kaffee. Der Kaffee schmeckt sehr gut und dazu passt am besten…
Abendessen mit Freunden	Du bist mit Freunden zum Abendessen verabredet. Nach dem Essen bist du satt und zufrieden. Du stehst auf und holst dir …
Ausgehen	Du bist auf einem geselligen Abend mit Freunden. Die Musik ist gut und die Stimmung ausgelassen. Du lehnst dich zurück und greifst nach …
**Negative/affective**	
Stress auf der Arbeit	Die Arbeit ist heute total stressig. Die Aufgaben wachsen dir über den Kopf und die Zeit drängt. Um dem Druck stand zu halten, machst du eine kurze Pause und …
Unordnung in der WG	Dein Mitbewohner richtet in eurer Wohnung ein riesen Chaos an. Als du ihn damit konfrontierst, kommt es zwischen euch zu einem Streit. Um den Kopf frei zu bekommen, gehst du aus der Wohnung und holst dir …

Lernen	Am Nachmittag musst du eine wichtige Klausur schreiben. Du bist schrecklich nervös und möchtest dich beruhigen. Jetzt hilft nur eins, und zwar …
Unfall	Ein Mitglied deiner Familie hatte einen schlimmen Autounfall. Nachdem du im Krankenhaus bei ihm warst, bist du voller Sorge. Auf dem Weg nach draußen willst du nur eins, nämlich …
Ein langer Tag	Dein Tag startet schlecht und wird immer schlimmer. Am Abend kommst du müde und frustriert nach Hause. Das Erste, was du machst, ist …
Erschöpfung	Der Vormittag hat dich ganz schön gestresst, sodass du jetzt ziemlich kaputt bist. Du brauchst etwas, um wieder in Schwung zu kommen. Dir fällt da nur eine Sache ein, nämlich …
Einschlafschwierigkeiten	Du kannst nicht schlafen, weil sich dein ganzer Körper ruhelos anfühlt. Du brauchst etwas, das dich beruhigt, etwas das sofort hilft. Du denkst: Das kann jetzt nur eins sein, und zwar...
Schlechte Laune	Dein bester Freund kommt zu Besuch. Er merkt schnell, dass du schlechte Laune hast. Da er dich gut kennt, reicht er dir zur Aufmunterung …
**Habit/addictive**	
Im Biergarten	Weil schönes Wetter ist, sitzt du mit Bekannten in einem Biergarten. Nachdem ihr eure Bestellung aufgegeben habt, unterhaltet ihr euch angeregt. Während ihr auf euer Essen wartet, greifst du nach …
An der Kasse	Du hast im Supermarkt für die Woche eingekauft. Nun stehst du an der Kasse und räumst die Sachen aus deinem Einkaufswagen. Nachdem alles auf dem Band liegt, ergänzt du deinen Einkauf noch um …
Warten auf den Bus	Du willst mit dem Bus in die Stadt fahren. Ausgerechnet heute hat er Verspätung. Während du an der Bushaltestelle stehst und wartest, vertreibst du dir die Zeit mit …
Nach dem Aufwachen	Der Wecker klingelt zur selben Uhrzeit wie immer. Du bist noch etwas müde und stehst langsam auf. Das erste, woran du denkst, ist …
Nach dem Termin	Du hattest einen wichtigen Termin, bei dem du lange Zeit in einem Büro gesessen hast. Endlich ist der Termin vorbei und du gehst nach draußen. Du freust dich an der frischen Luft zu sein und holst aus deiner Tasche …

English Translation:

Scenario type/Title:	Scenario
**Positive/social**	
A day at the lake	It's a lovely day and you are lying next to a beautiful lake. While you are relaxing, you think there's just one thing missing. So you reach into your bag and ...
Drinking beer	You are in a pub with friends. You are chatting and drinking a few bottles of beer. You realize that as you drink, you desire to ...
Before the disco	You are waiting in line at a disco with a few friends. One of them turns to you and asks "Do you want one?". You say yes and take ...
On the way to a party	You are on your way to a party. On the way there, you realize that you have forgotten something. So you run to the nearest kiosk and buy ...
Drinking coffee	You meet up with a friend you haven't seen for a while. While you talk, you have a cup of coffee. The coffee tastes very good and goes best with ...
Dinner with friends	You have a dinner date with friends. After dinner, you are full and satisfied. You get up and get yourself ...
Going out	You are on a social evening with friends. The music is good and the atmosphere is exuberant. You lean back and reach for ...
**Negative/affective**	
Stress at work	Work is very stressful today. The tasks are getting on top of you and time is running out. To cope with the pressure, you take a short break and ...
Mess in your apartment	Your roommate makes a huge mess in your apartment. When you confront them, you and they end up in an argument. To clear your head, you leave the apartment and get yourself a ...
Studying	You have to write an important exam in the afternoon. You are terribly nervous and want to calm down. There is only one thing to do now, and that's ...
Accident	A member of your family has had a bad car accident. After visiting them in hospital, you are full of worry. On the way out, you only want one thing, namely ...

A long day	Your day starts badly and gets worse and worse. In the evening, you come home tired and frustrated. The first thing you do is ...
Exhaustion	The morning has really stressed you out, so you are pretty exhausted now. You need something to get you going again. The only thing you can think of is ...
Sleep difficulties	You cannot sleep because your whole body feels restless. You need something to calm you down, something that helps immediately. You think: There can only be one thing right now, and that is...
Bad mood	Your best friend comes to visit. They quickly realize that you are in a bad mood. Since they know you well, they give you a drink to cheer you up.
**Habit/addictive**	
At the beergarden	Because the weather is nice, you are sitting in a beer garden with friends. After you have placed your order, you chat animatedly. While you are waiting for your food, you reach for a …
At the checkout	You have done your shopping for the week at the supermarket. Now you are at the checkout and take the items out of your shopping cart. After everything is on the conveyor belt, you add ...
Waiting for the bus	You want to take the bus into town. Today, of all days, the bus is running late. While you stand at the bus stop and wait, you pass the time with ...
After waking up	The alarm clock rings at the same time as always. You are still a little tired and get up slowly. The first thing you think about is ...
After an appointment	You had an important appointment where you sat in an office for a long time. Finally, the appointment is over and you go outside. You are happy to be out in the fresh air and take something out of your bag, namely ...

Neutral scenarios:

Please note that neutral scenarios were used as fillers and were not included to the main analysis.

German Version:

Scenario type/Title	Scenario
**Neutral**	
Ein langweiliger Film	Du machst mit deinen Freunden einen Filmabend. Der Film ist langweilig, also gehst du online. Im Internet fängst du direkt an zu …
	Du hängst noch ein wenig ab, bevor du in die Stadt gehst. Die Frage ist: Einen Film schauen oder am Computer spielen? Nachdem du eine Weile unsicher warst, entscheidest du dich für …
Snacks für das Kino	Du gehst mit deinen Freunden ins Kino. An der Theke holst du dir noch einen Snack. Heute Abend hast du mal Lust auf …
Auf dem Konzert	Du bist mit Freunden auf einem Konzert. Der Laden ist voll und die Musik ist laut. Sich mit deinen Freunden zu unterhalten, findest du sehr …
Klausurergebnisse	Du besuchst eine Vorlesung gemeinsam mit deinen Freunden. Dir wird gesagt, dass die Ergebnisse eurer Klausur bekannt gegeben wurden. Du checkst das Schwarze Brett, du hast …
Im Café	Du triffst dich mit einer alten Freundin im Café. Zu deiner Waffel hast du dir heiße Kirschen bestellt. Du findest, die Kirschen schmecken sehr …
Sport mit Freunden	Du möchtest dich mit Freunden sportlich betätigen. Zur Auswahl stehen Fußball und Volleyball. Heute hättest du Lust auf …
Nach dem Feiern	Du und deine Freunde seid ausgegangen. Ihr macht schnell Halt an einer Dönerbude. Du hast wirklich Lust auf …
Xbox	Du und deine Mitbewohner habt euch eine neue Xbox gekauft. Sie funktioniert nicht, also bringst du sie zurück zum Geschäft. Als der Verkäufer deine Geschichte hört, reagiert er sehr ...
Der nächste Tag	Du hast gerade eine Geburtstagsparty in deiner Wohnung geschmissen. Jetzt musst du alles wieder an seinen Platz zurückräumen. Die Wohnung aufzuräumen ist sehr …

Neue Wandfarbe	Du streichst deine Wohnung. Die Wand im Wohnzimmer soll eine schöne Farbe bekommen. Nach langem Überlegen entscheidest du dich für …
Reisepläne	Du möchtest mal wieder Verreisen. Eine Bekannte schlägt dir Paris als Reiseziel vor. Diesen Vorschlag findest du …
Zusammen lernen	Du und dein Studienkollege arbeitet an einer Seminaraufgabe. Dir fällt auf, dass ihr zwei sehr verschieden arbeitet. Im Vergleich zu ihm, bist du sehr …
Schlange stehen im Supermarkt	Vor dir an der Kasse steht ein Mann mit einem vollen Einkaufswagen. Da du nur drei Teile hast, fragst du ihn, ob er dich vorlassen würde. Auf deine Frage hin reagiert der Mann sehr …
Taxi	Du hast dir ein Taxi bestellt, das dich zum Flughafen fahren soll. Du wartest und hast die Zeit aus den Augen verloren. Du guckst auf die Uhr und …
Probierhäppchen im Supermarkt	Im Supermarkt steht ein Verköstigungsstand. Die Werbedame bietet dir einen milden und einen würzigen Käse an. Du betrachtest die beiden Proben und greifst nach …
Ein Spieleabend mit „Risiko“	Jedes zweite Wochenende spielst du ‚Risiko‘ mit deinen Freunden. Alles ist aufgebaut und die Missionen werden verteilt. Dieses Mal ist deine Mission sehr …
Eine neue Klasse	Du hast den Studiengang gewechselt. Alle kennen sich bereits, also stellst du dich vor. Deine neuen Kommilitonen finden dich bestimmt …
Hund eines Bekannten	Du machst einen Spaziergang und triffst zufällig einen Bekannten mit seinem Hund. Der Hund des Bekannten springt dich zur Begrüßung an. Du findest das Anspringen …
Hausparty	Du und dein Mitbewohner organisiert eine Hausparty. Jeder kann 5 Leute einladen. Du findest das Erstellen einer Einladungsliste sehr …

English Translation:

Scenario type/Title	Scenario
**Neutral**	
A boring movie	You are having a movie night with your friends. The movie is boring, so you go online. On the Internet, you start to ...
	You hang out for a while before going into town. The question is: watch a movie or play on the computer? After being unsure for a while, you decide to ...
Snacks for the movies	You go to the movies with your friends. You grab a snack at the bar. Tonight you fancy a ...
At the concert	You are at a concert with friends. The place is packed and the music is loud. You find talking to your friends very ...
Test results	You attend a lecture with your friends. You are told that the results of your exam have been announced. You check the notice board, you have ...
In a café	You meet up with an old friend in a café. You have ordered hot cherries to go with your waffle. You think the cherries taste very ...
Sports with friends	You want to play sports with friends. You can choose between soccer and volleyball. Today you would like to ...
After the party	You and your friends have gone out. You make a quick stop at a kebab stand. You really fancy a ...
Xbox	You and your roommates have bought a new Xbox. It does not work, so you take it back to the store. When the salesperson hears your story, they react very ...
The next day	You have just thrown a birthday party in your apartment. Now you have to put everything back in its place. Tidying up the apartment is very ...
New wall color	You are painting your apartment. You want to give the wall in the living room a nice color. After much deliberation, you decide on ...
Traveling plans	You want to go on a trip again. A friend suggests Paris as a destination. You find this suggestion ...
Learning together	You and your fellow student are working on a seminar assignment. You notice that the two of you work very differently. Compared to them, you are very ...

Queuing at the supermarket	There is a man in front of you at the checkout with a full shopping cart. As you only have three items, you ask him if he would let you go first. The man reacts to your question very ...
Cab	You have ordered a cab to take you to the airport. You are waiting and have lost track of time. You look at your watch and ...
Tasting stand in the supermarket	There is a tasting stand in the supermarket. The saleswoman offers you a mild and a spicy cheese. You look at the two samples and reach for ...
A games night with "Risk"	Every other weekend you play 'Risk' with your friends. Everything is set up and the missions are distributed. This time your mission is very ...
A new course	You have changed courses. Everyone else already knows each other, so you introduce yourself. Your new fellow students are sure to find you ...
A friend's dog	You are out for a walk and happen to meet a friend with his dog. The friend's dog jumps up to greet you. You find the jumping up ...
Houseparty	You and your roommate organize a house party. Everyone can invite 5 people. You find creating an invitation list very ...
